# The British Army as a Fighting Force

**Published:** 1896-02-22

**Authors:** 


### THE BRITISH ARMY AS A FIGHTING FORCE.

The Annual Report of the Army Medical Department, just issued as a Blue Book, deals among other matters with a subject sufficiently unsavoury to lead many people to pass it on one side, but one at the same time of such national importance as to oblige us to draw attention to it; an evil which not only hampers our army in its every movement, by burdening it with a crowd of invalids, but cripples it by spreading the taint of invalidism through the ranks of those who are supposed to be efficient. We allude to the enormous prevalence of venereal disease.

The average strength of the British army serving in the United Kingdom in 1894 was 99,360, and out of this number there were during the year 18,122 admissions for venereal disease, while the average number constantly sick was 1,632·06; that is to say, that in the United Kingdom there are always about two battalions laid up with these diseases, or 16·42 men in every thousand. This is very bad, but the condition of the army on foreign stations is far worse, and worst of all is the state of affairs in India, for not only is the expense of supporting English soldiers abroad very considerable, but the integrity of our foreign possessions depends upon the fighting efficiency of every man. For every thousand soldiers that we have in India, five hundred were admitted during the year 1894 suffering from venereal disease. Our Indian army is not a large one, and on its efficiency as a fighting force depends our hold upon that great country; yet from this handful of men, on whom the English women and children scattered all over its vast area depend for their safety, there is a permanent body of 1877·7 sick to be deducted, all of whom are incapacitated by venereal disease. Two large battalions, transported to India at enormous cost, maintained there regardless of expense, provided with every protection that science can devise against every disease except those of one description, are all the year round laid low by that particular class of malady and rendered useless to the country which pays so heavily for their services. That over 35,000 cases of venereal disease should occur in one year among a force of 70,000 men is an appalling fact. It is unnecessary to say how grave a condition of affairs is here displayed, nor need one hesitate to say that the continued prevalence of such an infectious disease is contrary to the spirit of all modern sanitary progress. If there is one point in regard to which sanitary science can justly boast it is in regard to the diminution in the prevalence of infectious diseases, which has been produced by carefully-devised sanitary measures. For the sake of these results the country has put up with much interference with individual liberty. Yet all the while we allow the existence unchecked in our midst of an infectious disease which not only spoils the efficiency of our army but lowers the vitality of the whole population, a disease, moreover, the dissemination of which depends upon one particular class of women, and is by them disseminated not as an accidental occurrence but purely and entirely as part and parcel of their daily trade, a trade which in itself is looked upon as a disgrace and a social evil. It is a strange and unaccountable condition of affairs that any nation which takes pride in its sanitary progress should allow free trade in vice to continue as it does, seeing all that it involves in the way of free trade in infection.

To understand what is going on under the present system of free trade in infection we must read these army reports. There is hardly one man in three out of the whole British army in India who has not been infected. The figures relating to secondary syphilis show to what an extent the army suffers from a disease which seriously undermines a man's constitution, makes him liable to break down at any time, particularly when exposed to the fatigues and hardships of active service, and at all times makes him less able to contend against acute disease. These are the men on whom we have to depend for the safety of our empire, and we take it that it is a satire both on sanitation and on common sense that while we are agitating ourselves about obscure questions regarding drainage and about doubtful problems in tuberculosis, we should allow our army, consisting of fresh-faced lads newly recruited from country homes, and on whose vigour and courage we depend, to be surrounded everywhere by a horde of diseased women—harpies who, as a mere trade, not only prey upon them and squeeze out of them the little money they may possess, but turn them out infected with diseases which undermine their strength and their vitality, and leave them fitted only for the life of sandwich men, to which they ultimately turn, instead of remaining what these lads all hoped to be when they enlisted—a fighting force on which their country might depend in her hour of danger.

